# Emerging Diagnostic Approaches for Musculoskeletal Disorders: Advances in Imaging, Biomarkers, and Clinical Assessment

**DOI:** 10.3390/diagnostics15131648

**Published:** 2025-06-27

**Authors:** Rahul Kumar, Kiran Marla, Kyle Sporn, Phani Paladugu, Akshay Khanna, Chirag Gowda, Alex Ngo, Ethan Waisberg, Ram Jagadeesan, Alireza Tavakkoli

**Affiliations:** 1 Department of Biochemistry and Molecular Biology, University of Miami Miller School of Medicine, Miami, FL 33136, USA; rxk641@miami.edu (R.K.); gowdachirag24@gmail.com (C.G.); axn668@med.miami.edu (A.N.); 2 Carver College of Medicine, University of Iowa, Iowa City, IA 52242, USA; kmarla@uiowa.edu; 3 Norton College of Medicine, Upstate Medical University, Syracuse, NY 13210, USA; spornk@upstate.edu; 4 Brigham and Women’s Hospital, Harvard Medical School, Boston, MA 02115, USA; phani.paladugu@students.jefferson.edu; 5 Sidney Kimmel Medical College, Thomas Jefferson University, Philadelphia, PA 19144, USA; aya156@students.jefferson.edu; 6 Department of Clinical Neurosciences, University of Cambridge, Cambridge CB2 3EH, UK; 7 Whiting School of Engineering, Johns Hopkins University, Baltimore, MD 21218, USA; ramjagad@cisco.com; 8 Department of Computer Science, University of Nevada, Reno, NV 89557, USA; tavakkol@unr.edu

**Keywords:** musculoskeletal imaging, biomarkers, clinical assessment, point-of-care ultrasound, artificial intelligence, movement analysis

## Abstract

Musculoskeletal (MSK) disorders remain a major global cause of disability, with diagnostic complexity arising from their heterogeneous presentation and multifactorial pathophysiology. Recent advances across imaging modalities, molecular biomarkers, artificial intelligence applications, and point-of-care technologies are fundamentally reshaping musculoskeletal diagnostics. This review offers a novel synthesis by unifying recent innovations across multiple diagnostic imaging modalities, such as CT, MRI, and ultrasound, with emerging biochemical, genetic, and digital technologies. While existing reviews typically focus on advances within a single modality or for specific MSK conditions, this paper integrates a broad spectrum of developments to highlight how use of multimodal diagnostic strategies in combination can improve disease detection, stratification, and clinical decision-making in real-world settings. Technological developments in imaging, including photon-counting detector computed tomography, quantitative magnetic resonance imaging, and four-dimensional computed tomography, have enhanced the ability to visualize structural and dynamic musculoskeletal abnormalities with greater precision. Molecular imaging and biochemical markers such as CTX-II (C-terminal cross-linked telopeptides of type II collagen) and PINP (procollagen type I N-propeptide) provide early, objective indicators of tissue degeneration and bone turnover, while genetic and epigenetic profiling can elucidate individual patterns of susceptibility. Point-of-care ultrasound and portable diagnostic devices have expanded real-time imaging and functional assessment capabilities across diverse clinical settings. Artificial intelligence and machine learning algorithms now automate image interpretation, predict clinical outcomes, and enhance clinical decision support, complementing conventional clinical evaluations. Wearable sensors and mobile health technologies extend continuous monitoring beyond traditional healthcare environments, generating real-world data critical for dynamic disease management. However, standardization of diagnostic protocols, rigorous validation of novel methodologies, and thoughtful integration of multimodal data remain essential for translating technological advances into improved patient outcomes. Despite these advances, several key limitations constrain widespread clinical adoption. Imaging modalities lack standardized acquisition protocols and reference values, making cross-site comparison and clinical interpretation difficult. AI-driven diagnostic tools often suffer from limited external validation and transparency (“black-box” models), impacting clinicians’ trust and hindering regulatory approval. Molecular markers like CTX-II and PINP, though promising, show variability due to diurnal fluctuations and comorbid conditions, complicating their use in routine monitoring. Integration of multimodal data, especially across imaging, omics, and wearable devices, remains technically and logistically complex, requiring robust data infrastructure and informatics expertise not yet widely available in MSK clinical practice. Furthermore, reimbursement models have not caught up with many of these innovations, limiting access in resource-constrained healthcare settings. As these fields converge, musculoskeletal diagnostics methods are poised to evolve into a more precise, personalized, and patient-centered discipline, driving meaningful improvements in musculoskeletal health worldwide.

## 1. Introduction

Musculoskeletal disorders represent one of the leading causes of disability worldwide, posing significant diagnostic challenges for clinicians because of their complex pathophysiology and heterogeneous presentation [1]. However, recent advances in diagnostic methodologies have revolutionized our ability to detect, characterize, and monitor these conditions [2,3,4]. This comprehensive review synthesizes current evidence on emerging diagnostic approaches across advanced imaging techniques, novel biomarkers, clinical assessment frameworks, artificial intelligence applications, and point-of-care technologies, among other frameworks [5,6,7,8,9]. By integrating these modalities, clinicians can detect novel pathologies earlier, stratify diseases more accurately, and facilitate more personalized treatment planning.

## 2. Evolution of Imaging Modalities in Musculoskeletal Diagnostics

### 2.1. Conventional Imaging: Strengths and Limitations

Conventional radiography remains the gold standard of initial musculoskeletal imaging due to its accessibility, relatively low cost, and ability to visualize bone architecture [10]. However, it is constrained by limited soft tissue contrast and the two-dimensional representation of three-dimensional structures [11]. Radiographs are excellent for evaluating fractures, joint space narrowing, osteophyte formation, and bone density changes, but can fail to detect early degeneration of cartilage or subtle soft tissue abnormalities [12,13]. These limitations are particularly evident in early-stage osteoarthritis, where significant cartilage deterioration may occur before clinicians may notice changes on radiographic findings [14,15].

Comparatively, computed tomography (CT) offers significant advantages over conventional radiography through its cross-sectional imaging capabilities and superior contrast resolution [16]. Recent technological advances, including photon-counting detector CT (PCD-CT), have enhanced spatial and contrast resolution compared with multidetector CT (MDCT), despite reductions in radiation exposure [17]. PCD-CT, in particular, is a significant innovation in CT imaging [18]. This new detector technology allows X-rays to be converted directly into an electrical signal without an intermediate step via a scintillation layer and enables measurement of the energy of individual photons [19]. PCD-CT can help clinicians visualize trabecular bone details and improve assessment of complex fractures as well as subtle osseous lesions [20,21]. The advantages of PCD-CT over conventional energy-integrating detector (EID) CT include smaller detector pixels, excellent geometric dose efficiency for high-resolution imaging of large joints and central skeletal anatomy, advanced multienergy spectral postprocessing, improved metal artifact reduction, and higher contrast-to-noise ratio with suppression of electronic noise [22,23,24].

However, the widespread clinical integration of PCD-CT is still limited by several factors. Studies have demonstrated its superior diagnostic accuracy in detecting fine bone structures and reducing artifacts in the presence of metal implants, while maintaining similar accuracy, sensitivity, and specificity in depicting cortical bone involvement and the number of bone lesions [25]. However, comparative studies on outcomes in MSK disease management remain limited. Furthermore, while PCD-CT can achieve higher image quality at lower radiation doses than EID-CT, the dose reduction varies by protocol and anatomical region, and standardized dose-to-benefit analyses for routine MSK imaging are still lacking [26,27,28]. The high capital cost of PCD-CT scanners, nearly five times the cost of conventional CT scanners, and the need for radiologists and technologists to undergo specialized training in spectral data interpretation present practical barriers to adoption [29,30]. Additionally, availability remains limited, with most systems currently restricted to major academic centers and tertiary care institutions [29]. Thus, while PCD-CT offers significant technical innovations with clear advantages over conventional CT in terms of image quality and tissue differentiation, broader clinical implementation will depend on continued validation of diagnostic impact, cost-effectiveness analyses, and expanded access through infrastructure investment and training initiatives.

Magnetic resonance imaging (MRI) has also transformed musculoskeletal diagnostics through its soft tissue contrast, multiplanar capabilities, and absence of ionizing radiation [31]. Practically, quantitative MRI techniques can provide noninvasive measures of cartilage degeneration at the earliest stages of joint degeneration [32,33]. These techniques can be categorized into those that grade and quantify morphologic changes and those that quantify changes in the extracellular matrix [34]. Morphological assessment of cartilage with quantitative MRI has demonstrated high accuracy and adequate precision in both cross-sectional and longitudinal studies in osteoarthritis patients (Table 1) [35,36,37].

### 2.2. Advanced Functional and Molecular Imaging

Another emerging technology with great promise for musculoskeletal uses in both clinical practice and research is four-dimensional computed tomography (4D-CT), often known as dynamic CT [38]. This method produces multiple CT measurements of a moving structure to show real-time motion [39]. Reduced radiation dose and recent developments in acquisition technology have enabled practical and safe imaging of joint motion [16,40] and greater acceptance of this modality. Wrist motion has been investigated using 4D-CT, and the method’s value has been shown in other anatomical areas like the shoulder, elbow, hip, knee, and ankle [41]. Imaging these joints during a whole range of motion offers fresh perspectives on dynamic events like joint kinematics, impingement, and instability [42]. Pilot studies using optical motion-capture methods to validate 4D-CT analysis of knee joint movement have demonstrated promising results [43]. Early comparisons between 4D-CT with 3D-3D registration and optical motion-capture systems suggest that 4D-CT provides high accuracy when capturing knee joint kinematics [44,45]. Overall, these preliminary investigations indicate that 4D-CT with 3D-3D registration may serve as a reliable tool for in vivo kinematic analysis in musculoskeletal research and clinical assessment (Table 1) [46].

Molecular imaging likewise offers fresh perspectives on the cellular and metabolic processes underlying disease pathogenesis [47]. Namely, fluorescence-based methods have become very effective tools for exploring bone biology and cellular activity in vivo [48]. For example, fluorescence-based assays for real-time myeloid cell to osteoclast development (FRAMCO) [49] have been created by scientists. By means of the red-to-green fluorescence conversion of certain transgenes controlled by osteoclast-specific promoters, these assays enable osteoclast-specific gene expression and intercellular fusing of pre-osteoclasts [50,51]. Comprehending bone metabolism across various clinical diseases and assessing the efficacy of therapeutic strategies targeting bone turnover significantly relies on the non-invasive monitoring of osteoblast and osteoclast activity [49,52].

### 2.3. Point-of-Care Ultrasound

Point-of-care ultrasound (POCUS) provides immediate, dynamic, and economical imaging options while avoiding ionizing radiation exposure to patients [53]. In emergency settings, POCUS has shown robust diagnostic capabilities for fracture detection, with numerous studies indicating elevated sensitivity and specificity metrics [54,55]. Pilot investigations indicate that POCUS can effectively identify various fracture types, suggesting its potential as an alternative to radiography for diagnosing and characterizing fractures, particularly in the emergency room (ER) [54,56,57]. Additional studies assessing the accuracy of POCUS in patients with suspected long bone fractures support its diagnostic value, with findings indicating that POCUS may decrease dependence on formal radiography depending on the specific clinical situations, thereby accelerating diagnosis and treatment processes [58].

In addition to detecting fractures, POCUS can help clinicians rapidly assess soft tissue injuries and subsequently perform ultrasound-guided interventional procedures to stabilize patients [59,60]. Ultrasound-guided corticosteroid injections for shoulder pathology, including arthritis and adhesive capsulitis, have shown favorable results in musculoskeletal care [61,62,63]. Educational initiatives highlight the significance of appropriate ultrasound-guided techniques, encompassing patient positioning, probe placement, and precise medication administration into target joints [64,65]. Clinical studies indicate that ultrasound guidance may improve therapeutic accuracy and enhance patient outcomes, especially in shoulder conditions such as adhesive capsulitis [66,67]. POCUS has also been used for rapidly evaluation of rotator cuff injuries when conventional radiography has provided conflicting findings [68,69]. Structured methods have been developed to standardize ultrasonic examinations of the shoulders [68] including the ABSIS (acromioclavicular joint, biceps tendon, subscapularis, impingement, supraspinatus) approach. Demonstrating great diagnostic accuracy for full-thickness tears and acting as a useful adjunct to clinical evaluation, ultrasonic imaging can clearly reveal important features of supraspinatus tendon tears and other rotator cuff disorders (Table 1) [70,71,72].

## 3. Biomarkers in Musculoskeletal Disease Stratification

### 3.1. Inflammatory and Bone Turnover Markers

Biomarkers are essential for assessing and monitoring musculoskeletal disorders, particularly those characterized by inflammatory or degenerative elements [73,74]. C-terminal cross-linked telopeptides of type II collagen (CTX-II) have been extensively studied biomarkers for osteoarthritis, especially in the knee [75], and are among the most frequently assessed and easily accessibly biomarkers for bone breakdown [76]. Increased urinary CTX-II levels have been consistently linked to the presence and progression of knee osteoarthritis, correlating with cartilage degradation and disease severity [77,78]. Emerging evidence indicates that differences related to sex and ethnicity may impact CTX-II expression patterns, potentially influencing biomarker performance in various patient populations [79]. The findings indicate that urinary CTX-II may effectively differentiate osteoarthritis patients from healthy individuals and offer insights into disease progression, though demographic factors are crucial for accurate interpretation [80].

However, it is important to note that this marker exhibits notable diurnal variability, with higher values in early morning that drop as the day progresses, although preliminary research suggests standardization can be achieved through consistent timing of sample collection (e.g., midday) to improve reproducibility [81,82]. Substantial inter-individual differences have been observed, partly influenced by demographic factors. For example, a meta-analysis reported that urinary CTX-II had greater discriminative power for knee osteoarthritis in women and in European cohorts compared with men and Asian cohorts [76]. Despite this variability, the biomarker’s prognostic relevance has been reproducibly demonstrated in large populations. In one cohort (~1235 subjects), individuals with the highest CTX-II levels had roughly a six-fold increased risk of radiographic knee OA progression over time, highlighting its potential for monitoring disease progression [83]. Currently, however, no cartilage degradation marker (including CTX-II) has yet been adopted into routine clinical practice or guidelines, as no single biomarker is considered sufficiently validated or reliable for standard use in osteoarthritis management (Table 2) [84]. Bone turnover markers (BTMs) are a valuable collection of biomarkers that provide pertinent insights on mechanisms of bone remodeling [85]. In evaluating the efficacy of osteoporosis treatment [86], clinicians often rely on serum procollagen type I N-propeptide (PINP), a sensitive indicator of osteoblast activity and a sign of novo collagen synthesis inside the bone matrix [87]. In fact, clinical guidelines for therapy response often ask for baseline PINP measurement and evaluation following osteoporosis treatment initiation [88,89]. Variations in PINP levels during medication may provide important new directions for treatment mechanism research [90]. Generally associated with lower levels of PINP, anti-resorptive treatments such as bisphosphonates indicate a decrease in bone turnover [91]. Teriparatide and other anabolic drugs increase bone production, which over time raises PINP levels [92]. Referring to these biomarker patterns, physicians can better distinguish therapeutic outcomes and customize osteoporosis treatment plans [93].

Although unlike CTX-II, PINP shows relatively low biological variability with regards to diurnal variation, seasonal fluctuations, and differences between sexes, there exist limitations to its clinical use. An important confounding factor is renal function; the monomeric form of PINP tends to accumulate in patients with advanced renal impairment, leading to spuriously high total PINP levels in chronic kidney disease. Using an assay that detects only intact (trimeric) PINP can mitigate this effect in CKD settings [94]. Clinically, PINP is recommended as a reference marker in osteoporosis management guidelines (endorsed by IOF/IFCC) for fracture risk assessment and for monitoring responses to therapy (Table 2).

### 3.2. Genetic and Epigenetic Biomarkers Associated with Disease Development

Genetic biomarkers can also help clinicians predict disease susceptibility, progression, and response to treatment [95]. The GDF5 gene, which encodes growth differentiation factor 5, contains a functional single nucleotide polymorphism (SNP), rs143383, that has been consistently associated with osteoarthritis development [96]. This C/T transition in the 5′ untranslated region (5′UTR) of the gene forms a CpG site in its C-allele form and mediates differential allelic expression of GDF5, with the disease-associated T allele demonstrating reduced expression [97]. The differential allelic expression imbalance of the C and T alleles varies intra- and inter-individually, suggesting that this effect may be modulated epigenetically [98]. Research has demonstrated that DNA methylation regulates GDF5 expression and the allelic imbalance caused by rs143383 [99]. The CpG sites created by the C alleles at rs143383 and a nearby SNP (rs143384) are variably methylated, and treatment of a heterozygous cell line with a demethylating agent further increased the allelic expression imbalance between the C and T alleles [100]. This finding demonstrates that the genetic effect of the rs143383 SNP on GDF5 expression is modulated epigenetically by DNA methylation [101]. The variability in differential allelic expression of rs143383 is therefore partly accounted for by differences in DNA methylation, which may influence the penetrance of this allele in arthritis susceptibility, as well as other common musculoskeletal diseases (Table 2) [102].

Despite its strong biological plausibility and mechanistic support, the translational relevance of the rs143383 SNP remains limited, although clinical trials for disease-modifying drugs are currently in progress [103]. While several studies have demonstrated a correlation between this polymorphism and osteoarthritis (OA), the effect size is modest. One large meta-analysis reported an odds ratio of 1.10 for the T allele, with the strongest associations observed in European populations, followed by Japanese cohorts [104,105]. Additionally, evidence suggests that the genetic contribution of rs143383 varies by OA site, with the most robust associations reported for hip and knee OA [95]. In vitro studies of joint tissues from OA patients carrying the T allele have demonstrated differential expression patterns involving downstream pathways, including altered binding affinity of the deformed epidermal autoregulatory factor 1 (DEAF-1), which may contribute to disease pathogenesis. However, these downstream molecular effects have not been well characterized across other joint sites, such as the hands or spine [97].

Additionally, limited data exist for Latin American and other underrepresented ethnic populations, underscoring the need for larger, multiethnic cohort validations. In fact, a lack of association with this SNP variant has been established among Greek Caucasians [106]. To date, no clinically validated cutoff values, sensitivity, specificity, or area under the receiver operating characteristic curve (AUC) have been established for rs143383 for diagnostic or prognostic use, limiting its current utility to research settings.

Such interactions between genetic and epigenetic elements highlight how musculoskeletal disease etiology can often be complex and thus can greatly benefit from combined genetic and epigenetic biomarketer panels for disease risk assessment and stratification [107]. Ideally, greater understanding of these molecular pathways will enable complex biomarketer panels to capture patients’ genetic susceptibility and epigenetic modifications, thus improving identification of persons or populations at high risk of negative musculoskeletal health outcomes [108,109].

### 3.3. Novel Biochemical Markers for Disease Monitoring

The terrain of biochemical indicators for musculoskeletal illnesses is changing as new technologies enable increasingly precise evaluation techniques [83]. Multiplex assays made possible by advanced molecular diagnostic technologies may simultaneously detect many biomarkers from low sample quantities, hence enhancing the efficiency and comprehensiveness of biomarketer profiling [110]. For the surveillance of complicated conditions such as rheumatoid arthritis and osteoarthritis, where many pathophysiological mechanisms coexist concurrently [111,112], these developments are particularly important. To improve the evaluation of joint health and disease activity, researchers are looking at markers of inflammation, cartilage degradation, and bone turnover [113,114,115,116]. A significant development in this sense is point-of-care testing for biochemical indicators, which provides laboratory-quality diagnostics in clinical settings [117]. Rapid testing systems greatly cut turnaround times from days to minutes, therefore enabling real-time clinical decision-making during patient visits [118]. Combining biomarketer evaluation with imaging results and clinical data fosters a complete approach for disease surveillance [119]. As biomarkers often detect molecular changes before they become apparent as observable structural abnormalities [120], the association of biomarker levels with imaging-detected structural changes provides much-needed complementary information on disease state.

## 4. Integrative Clinical Assessment Frameworks

### 4.1. Comprehensive Physical Examination Approaches

Clinicians include physical examinations in their assessments to better understand structural integrity, functional capacity, and pain generators, which cannot be fully captured by imaging or laboratory tests alone [121]. A systematic approach to physical examination is essential for accurate diagnosis and appropriate management planning [122]. This typically begins with visual inspection for asymmetries, deformities, muscle atrophy, or swelling, followed by palpation to identify areas of tenderness, temperature changes, effusions, or abnormal tissue texture [123]. Range-of-motion assessment, both active and passive, helps determine movement limitations and fragility, and whether these are induced by pain, stiffness, or mechanical blockage [124]. Neurovascular examination, including strength testing, sensory assessment, reflex evaluation, and vascular checks, complements this basic framework [125]. Special tests targeting specific pathologies constitute an essential component of physical examination, though their utility depends heavily on proper execution and interpretation within the clinical context [126]. These tests are designed to stress particular anatomical structures or reproduce specific symptoms, thereby helping to confirm or exclude suspected diagnoses [127]. For example, in shoulder assessment, tests like the Hawkins–Kennedy impingement test, Neer impingement sign, and empty can test help evaluate for rotator cuff pathology or impingement syndrome [128]. Similarly, in knee examination, the Lachman test, anterior and posterior drawer tests, and valgus/varus stress tests assess ligamentous integrity [129].

Functional assessment must also evaluate how patient conditions impact activities of daily living, occupational tasks, and recreation [130]. This typically involves observing the patient performing relevant movements or activities that provoke symptoms, revealing dysfunctional movement patterns, compensatory strategies, or activity-specific limitations that might not be apparent during standard examination maneuvers [131]. Standardized functional assessment tools provide objective measures of functional performance that can be tracked over time to evaluate treatment efficacy [131], which is particularly important for designing interventions that address not just structural abnormalities but also the resulting functional limitations [132].

### 4.2. Dynamic Assessment and Movement Analysis

Traditional static assessments often fail to capture the intricate coordination of muscle activation patterns, joint kinematics, and neuromuscular control required during functional movements [133]. Dynamic assessment overcomes this limitation by evaluating patients during active tasks, providing insights into compensatory strategies, movement asymmetries, and altered motor control that may underlie symptoms or predispose individuals to injury [134].

Objective assessment of gait using inertial measurement units (IMUs), wearable sensors combining accelerometers, gyroscopes, and magnetometers, has shown great promise for functional evaluations in individuals with knee osteoarthritis [135]. Among those with knee osteoarthritis, gait study using IMUs has repeatedly shown variations in spatiotemporal characteristics between patients and healthy controls [136]. Particularly indicating general deficits in locomotor efficiency, those with knee osteoarthritis often show shortened stride length, slower gait speed, and longer stride duration [137,138]. Furthermore, there was increasing variance in stride time, suggesting possible deficiencies in neuromotor control and gait stability [139].

Further analysis of joint and segmental kinematics reveals that individuals with knee osteoarthritis often exhibit reduced knee range of motion during the swing phase, diminished lumbar motion in the coronal plane, and altered foot strike and toe-off mechanics compared to healthy subjects [140]. These kinematic changes may represent adaptive strategies to minimize pain or mechanical loading on affected joints. Overall, inertial sensor technology offers a sensitive and accessible means of detecting mobility impairments in knee osteoarthritis, with spatiotemporal parameters emerging as particularly robust indicators of functional decline [141]. More detailed analyses of joint-specific kinematics, such as knee and trunk movements, may provide additional insights, although current evidence for these parameters is somewhat less consistent [142].

Technological developments have greatly improved the objectivity and accuracy of movement analysis, therefore moving it from subjective visual judgment to measurable measurement [143]. From laboratory-based optoelectronic systems to more portable inertial sensor-based technologies, motion capture systems provide thorough kinematic analysis of joint angles, velocities, and accelerations during functional activities [144]. Similarly, surface electromyography (sEMG) offers information regarding muscle activation patterns and time, therein aiding clinicians in understanding neuromuscular coordination impairments or changed recruitment tactics in response to discomfort or disease [145]. Ground response forces, center of pressure trajectories, and weight distribution patterns are quantified using force plates and pressure mapping devices both standing and walking [146]. Translating quantitative data into useful therapeutic insights depends critically on the junction of movement analysis results with clinical reasoning [147]. Observed movement anomalies should be understood in light of the patient’s anatomical structure, pain mechanisms, acquired habits, and psychological elements that could affect particular situations, including movement strategies [148]. For example, changed movement patterns might be a main cause driving symptoms, a subsequent adaptation to underlying illness, or a protective mechanism to prevent expected discomfort [149]. Differentiating these options calls for clinical judgment guided by thorough evaluation [150].

However, these emerging technologies still face important limitations that affect their clinical utility. Inertial sensors and sEMG systems can be impacted by calibration errors, sensor drift, signal noise, and soft-tissue/movement artifacts, all of which degrade measurement accuracy [151,152]. Additionally, although there exist disease-specific anatomical placement protocols of these devices, these are general guidelines that are not precise enough to overcome reproducibility errors [153]. There are no clear protocols regarding data filtering, making it difficult to compare results across studies and clinics. For example, in sEMG, electrode placement variations of just 2–3 cm can lead to signal amplitude changes of up to 50% and altered muscle activation pattern [154]. Wearable IMUs face similar calibration issues, with a compounding issue of orientation in addition to location placement [155]. Although some studies show accceptable data reliabilty for single IMU and sEMG device operators [156], differences in how clinicians place sensors or interpret movement data can lead to measurable discrepancies in the captured metrics, an issue which has been cited as major source of uncertainty in gait outcomes [157].

### 4.3. Integration of Patient-Reported Outcomes

Patient-reported outcome measures (PROMs) can also provide important insights into musculoskeletal disorders, namely by directly recording patients’ views of their symptoms, functional capacities, and quality of life [158]. Standardized questionnaires organize these self-reports, thereby augmenting objective results [159] with clinician-based evaluations. Additionally, they target symptoms and functional limitations related to certain disorders. For instance, the disease-specific instruments such as the Western Ontario and McMaster Universities Osteoarthritis Index (WOMAC) for osteoarthritis and the Roland–Morris Disability Questionnaire for low back pain [160]. Tools tailored to certain anatomical locations, such as the Lower Extremity Functional Scale (LEFS) and the Disabilities of the Arm, Shoulder and Hand (DASH) questionnaire, evaluate functionality in those areas from the patient’s perspective [161]. More general health-related quality of life across a variety of patient groups may be assessed by broader health status assessments such as the Short Form-36 (SF-36) and EuroQol-5D (EQ-5D [162]).

PROMs must also be evaluated for their psychometric qualities, including validity, reliability, responsiveness, and interpretability [163]. While consistency over repeated administrations guarantees dependability, validity guarantees that the instrument measures its intended construct. Interpretability highlights the clinical relevance of score fluctuations [164], whereas responsiveness measures the sensitivity of the instrument to clinically significant changes. Many widely used musculoskeletal PROMs [165] have scientific legitimacy due to such extensive validation efforts. For numerous instruments, minimal clinically important difference (MCID) values have been developed so that clinicians can determine whether the noted changes indicate significant patient improvement [166,167].

Practically, PROMs have shown to streamline data collecting, lower administrative load, and make possible instantaneous scoring and visualization through electronic administration on tablets, cellphones, or web-based platforms [168]. Logically, therefore, including PROMs into clinical procedures will improve the availability of patient-reported data, hence promoting better informed and prompt decision-making [169,170].

## 5. Artificial Intelligence and Decision Support Systems

### 5.1. Machine Learning Applications in Imaging Interpretation

Machine learning (ML) is significantly enhancing musculoskeletal imaging and increasing precision by automating disease diagnosis, categorization, and quantification [171]. Particularly among many imaging modalities including radiography, MRI, and ultrasonic techniques [172], convolutional neural networks (CNNs), and deep learning models have shown remarkable performance. Studies into cascaded and progressive CNN architectures that have evaluated models for successively detecting the meniscus and describing the tear form have shown that these models demonstrate clinician-similar efficacy in MRI interpretation [173,174]. Although these models demonstrate great accuracy in meniscus localization and overall tear classification, research indicates that discrimination between tear orientations is still underdeveloped [173,175]. More recent studies, meanwhile, have looked at how CNNs can improve meniscus tear characterization [151]. Modern approaches give the site of injury initial priority before categorizing tear types as horizontal, complicated, radial, or longitudinal arrangements. Although accuracy varies across different tear topologies [174,176], the findings show notable model efficiency in identifying medial and lateral meniscus lesions. Comparative studies show that, in select cases, machine learning models—especially deep convolutional neural networks (DCNNs)—can reach diagnostic performance on par with that of experienced radiologists.

Recent studies assessing AI imaging analysis further support this finding. AI-guided detection of distal radius fractures has demonstrated diagnostic accuracy and F1 scores (0.947) nearly identical to those of experienced orthopedic surgeons [177]. Similarly, systematic reviews and meta-analyses evaluating AI performance in femoral neck fracture assessment and leg axis measurements report excellent accuracy and specificity, with strong agreement between AI and human raters across large datasets [178,179]. These findings reinforce the growing reliability of AI in musculoskeletal imaging tasks traditionally reliant on expert interpretation.

Limitations affecting CNNs include data bias, lack of generalizability, and regulatory issues. Algorithms trained on imbalanced datasets (for example, over-representing certain demographics or single-institution images) can produce skewed outputs and perform unreliably on underrepresented patient groups. This is a cited problem with large datasets [180,181]. This lack of diversity and representativeness in the data also undermines generalizability, as evidence by substantial drops in performance on external dataset models following high performance on internal test sets. These drops in external dataset performance may be attributed to the data including unseen populations or imaging devices from different institutions and vendors [182,183]. Additionally, as of 2023, only a single fracture-detection (Gleamer BoneView) CNN AI tool has FDA clearance for broad clinical use, reflecting the stringent evidence requirements for safety and efficacy [181]. Even after approval, current regulations hinder adaptability; for instance, FDA-cleared AI models cannot be substantially modified or retrained without new certification, limiting their ability to correct biases or adapt to evolving imaging protocols [183].

Clinicians must recognize that practical variability in imaging interpretation is heavily influenced by both the anatomical location and the complexity of lesion categorization [184,185,186,187,188]. At the same time, modern algorithms have advanced significantly, enabling simultaneous measurement of multiple imaging biomarkers, assessment of disease severity through established clinical grading systems, and identification of anomalies and lesions using segmentation or object detection techniques [189]. These innovations increasingly mirror the demands of clinical decision-making, where precise and comprehensive imaging assessments are critical for prognostic evaluations [190].

### 5.2. Predictive Analytics for Disease Progression

Predictive analytics has become an increasingly powerful tool for forecasting disease trajectories in musculoskeletal disorders, providing clinicians and patients with actionable insights for treatment planning and prognosis [191]. By harnessing large datasets and advanced machine learning techniques, predictive models can reveal complex relationships and patterns that traditional clinical assessments often overlook [192]. Recent applications highlight the ability of machine learning algorithms to outperform standard predictive tools in estimating critical clinical outcomes, such as postoperative recovery metrics in musculoskeletal procedures [193,194,195]. Key patient factors—including demographic, functional, and socioeconomic variables—frequently emerge as important predictors, emphasizing the multifaceted nature of musculoskeletal care [196,197,198]. These developments illustrate the potential for healthcare systems to tailor predictive models to their specific populations and settings, thereby enhancing clinical decision-making beyond reliance on generalized public tools [199]. As predictive modeling evolves, diverse machine learning methods including random forests, support vector machines, and neural networks have been employed to anticipate disease progression, predict treatment responses, and assess complication risks [200,201]. Unsupervised approaches, such as clustering algorithms, further contribute by identifying distinct patient phenotypes and enabling more personalized management strategies [202]. This expanding methodological landscape empowers researchers and clinicians to align predictive models with specific clinical needs and available data, ultimately improving the precision, relevance, and impact of musculoskeletal care [203].

### 5.3. Clinical Decision Support Systems in Practice

As they become increasingly incorporated into clinical practice, clinical decision support systems (CDSSs) provide evidence-based advice at the time of care to improve diagnostic accuracy and treatment planning [204,205]. Practically, a CDSS may provide customized suggestions that consider patient genetics, demographics, and lifestyle and compare this data against accepted clinical guidelines, active, ongoing research, and historical results [206]. In turn, these CDSSs can aid physicians by recommending appropriate imaging, bloodwork, and other tests [207]. Despite their transformative potential, the operationalization and sustained integration of CDSSs into clinical workflows face substantial systemic and technical barriers [208,209,210]. Chief among these challenges is alert fatigue, wherein the high frequency or low specificity of system-generated notifications leads clinicians to disregard or override alerts, thereby attenuating system utility [211]. To mitigate this fatigue, developers can begin optimizing alert thresholds, implementing tiered prioritization schemas, and designing context-aware, minimally disruptive user interfaces so that all clinicians, regardless of technology experience, can leverage these systems [212]. Additionally, as medicine is an active, dynamic field, researchers must continuously curate and update CDSS knowledge bases, and this requires structured methodologies for assimilating emerging clinical evidence into actionable algorithmic outputs [213]. The reliability and predictive validity of CDSS outputs are thereby fundamentally contingent upon the integrity, standardization, and completeness of the underlying clinical data inputs. In the context of hospital administration, this underscores how important robust data governance frameworks, interoperable data architectures, and systematic validation protocols can be for clinical outcomes [214].

Unfortunately, like CNNS, CDSS also face issues of generalizability. Numerous decision tools used in clinical trials in the UK have shown only small benefits in controlled studies and negative results according to validation studies, implying the lack of generalizability and application to multicenter environments [215]. There is an additional barrier to implementation from physicians themselves who report negative perceptions, citing data bias and poor accuracy, probably due to insufficient data [216]. Finally, data privacy and security concerns remain issues because CDSSs depend on sensitive patient data, while strict privacy regulations (such as HIPAA in the US or GDPR in Europe) heavily restrict data sharing and aggregation, which may prevent the development and continuous improvement of these systems [217].

## 6. Challenges and Future Directions

### 6.1. Standardization and Validation Requirements

In musculoskeletal diagnostics, standardizing diagnostic modalities is a key requirement for improving imaging acquisition methodologies and biomarker reference ranges [218]. Variations in image acquisition settings, equipment standards, and post-processing procedures may have a significant impact on diagnostic accuracy and longitudinal comparability [219]. Similarly, variations in magnetic field strength, pulse sequences, slice thickness, and contrast administration procedures in MRI limit cross-center research and the development of uniform diagnostic criteria [220].

The clinical relevance of biomarkers is dependent on the establishment of reference standards and reliable measurement procedures [221]. Some biomarkers, such as PINP, have become highly standardized, with commercial tests yielding identical results in persons with normal renal capacity. Other biomarkers, on the other hand, vary depending on the analytical approach, sample processing, and collection methods [222]. The formulation of consensus guidelines by professional societies, the development of calibration standards, and the implementation of quality assurance programs are all important steps toward addressing standardization issues and enabling more consistent diagnosis and monitoring across different healthcare environments [223].

Before they can be implemented in routine clinical practice, novel diagnostic techniques must undergo extensive validation to verify that they significantly enhance diagnosis accuracy, patient outcomes, and healthcare economy. Clinical validation must begin with analytical validation, which ensures that the technology measures its intended parameters with sufficient accuracy and repeatability. The subsequent step is clinical validation, which involves the establishment of diagnostic performance criteria by defining sensitivity, specificity, and predictive values for relevant patient groups. Finally, validation of clinical utility demonstrates how the diagnostic method improves patient outcomes and facilitates clinical decision-making [224,225].

### 6.2. Integration of Multiple Diagnostic Modalities

Combining many diagnostic modalities is a strong approach to overcoming the restrictions of certain methods and enable more complete assessment of musculoskeletal disorders [226]. Every modality has different strengths and drawbacks; imaging clearly visualizes structural abnormalities but often misses functional abnormalities or molecular changes; laboratory biomarkers detect biochemical changes before structural damage but lack anatomical specificity; clinical assessments give functional insights but remain vulnerable to examiners’ subjectivity and variability [227].

By means of systematic integration of these complimentary techniques, the processes of illness may be better understood, early and more accurate diagnosis is made possible, disease stratification can be improved, and more focused treatment strategies are generated [228]. In complicated presentations including chronic knee pain, combining modern MRI methods (e.g., T1rho mapping for cartilage composition), inflammatory biomarkers, and thorough movement analysis may reveal pathogenic patterns unseen to any one modality [229]. Reducing needless testing and establishing standardized multimodal procedures that specify the most effective diagnostic combinations for certain clinical settings may help to increase diagnosis accuracy and efficiency [230].

In the application of artificial intelligence and advanced statistical modeling to detect patterns across imaging, biomarkers, clinical parameters, genetic data, and patient-reported outcomes that conventional analysis may overlook, computational integration of multimodal data represents a fundamental advance [35]. Synthesizing these varied inputs, machine learning techniques may find new disease subgroups and prognostic signals exceeding conventional diagnostic categories [231]. For characterizing the complex, multivariate nature of musculoskeletal diseases, where single biomarkers or imaging characteristics seldom provide adequate diagnostic or prognostic accuracy [232], computational techniques are becoming indispensable.

### 6.3. Ethical Considerations and Cost-Effectiveness

Clinicians must consider ethical issues including fair access, informed permission, handling of accidental results, and privacy protection into account before deploying these tools into practice [233]. In particular, the development of diagnostic technology and AI tools marked by increasing complexity and expense may aggravate inequality in access [234]. One approach to address equity concerns is the development of reasonably priced substitutes such as point-of-care ultrasonic devices for underprivileged or rural areas [235]. Overdiagnosis is also another pertinent issue, particularly with highly sensitive imaging modalities that often find abnormalities in asymptomatic patients [236]. MRI investigations of asymptomatic volunteers often show disc bulges, rotator cuff rips, and meniscal abnormalities, which may be due to age or adaptation to disease [237,238]. The growing availability of direct-to-consumer imaging technologies, free of clinical supervision or contextual analysis, further worsens this issue, as patients may attempt to self-treat conditions that may have no disease basis [239].

Additionally, cost-effectiveness considerations have become increasingly critical in allocating healthcare resources, particularly regarding modern diagnostic technologies that offer only modest clinical advantages at substantially higher costs [240]. Economic evaluations must account not only for the direct costs of diagnostic tests but also for their downstream impact on treatment decisions, clinical outcomes, and overall healthcare utilization [241]. While advanced quantitative MRI techniques may demonstrate superior technical performance for early osteoarthritis detection compared with standard sequences, their clinical value ultimately depends on whether earlier diagnosis leads to interventions that modify disease progression and improve patient outcomes [242].

## 7. Conclusions

The fields of orthopedics, sports medicine, and musculoskeletal diagnostics are experiencing significant change, driven by technological advancements, computational progress, and enhanced understanding of disease mechanisms [243,244,245]. This review examines various aspects of this evolution, including emerging imaging modalities, molecular biomarkers, artificial intelligence applications, and point-of-care technologies [246]. Advanced MRI techniques provide comprehensive evaluations of tissue composition and metabolism, facilitating the identification of cartilage degeneration prior to observable structural alterations [247]. Molecular imaging offers exceptional insights into bone remodeling and inflammatory pathways [248]. Point-of-care ultrasound has enhanced real-time imaging availability in various clinical environments, exhibiting high diagnostic accuracy for multiple musculoskeletal conditions [249]. Novel biomarkers like CTX-II for osteoarthritis and PINP for bone turnover provide objective assessments of disease activity and therapeutic response [250]. Advancements in genetic and epigenetic profiling can reveal individual patterns of susceptibility and identify potential therapeutic targets [251].

A major change in musculoskeletal treatment is the use of digital technology, artificial intelligence, and machine learning in diagnosis processes [252]. Across several applications, including the automatic identification of meniscal tears on MRI and #prediction of length of hospital stay after joint replacement [253], machine learning models show strong performance. With their evidence-based recommendations at the point of treatment, clinical decision support systems help to minimize clinical variability and enhance adherence to recommended practices [254]. By allowing real-world continuous evaluation of symptoms, functioning, and treatment response, mobile platforms and wearable sensors improve diagnosis monitoring [255].

Despite these advancements, considerable challenges persist with regard to converting diagnostic innovations into quantifiable enhancements in patient outcomes [256]. Standardization of imaging protocols, biomarker assays, and clinical assessment methods is essential for achieving consistent diagnoses and reliable longitudinal monitoring [257]. Validation studies should rigorously establish both technical performance and measurable effects on clinical decision-making and outcomes [258]. Integration of structural, functional, and molecular data has the potential to enhance disease characterization; however, it requires advanced data harmonization strategies and effective visualization tools [259]. Addressing these challenges necessitates collaborative efforts among researchers, clinicians, industry leaders, regulatory agencies, and healthcare systems [260]. As these efforts advance, it is very likely that musculoskeletal diagnostics will evolve toward more precise, personalized, and patient-centered methods for disease detection and monitoring [261].

## Figures and Tables

**Table 1:** Comparative Summary of Imaging Modalities in Musculoskeletal Diagnostics.

Imaging Modality	Diagnostic Accuracy	Implementation Cost	Availability
**MRI**	Excellent for soft tissue characterization, early cartilage degeneration, and quantitative assessment of extracellular matrix changes using advanced sequences.	High (equipment, operation, and maintenance)	Widely available in tertiary hospitals and outpatient imaging centers.
**CT**	Superior for high-resolution bone imaging, fracture assessment, and cortical abnormalities; limited for soft tissue or early cartilage degeneration.	Moderate to high (depends on detector type; PCD-CT higher)	Common in hospitals; PCD-CT systems limited to academic/tertiary care centers due to high cost.
**4D-CT**	High accuracy for in vivo joint kinematics and dynamic assessment of joint motion (e.g., impingement, instability); validated against optical tracking systems.	Very high (advanced acquisition hardware and processing)	Limited to research institutions or specialized centers with advanced motion analysis capability.
**POCUS**	Moderate to high sensitivity for fracture detection; effective for soft tissue assessment (e.g., rotator cuff tears) and image-guided injections; operator-dependent.	Low (portable, minimal infrastructure required)	Widely accessible in emergency departments, sports medicine, and outpatient clinics.

**Table 2:** Biomarkers in Musculoskeletal Disease: Biological Roles, Diagnostic Utility, and Clinical Readiness.

Biomarker	Biological Role	Clinical Context	Strengths	Limitations	Clinical Readiness
**CTX-II** (C-terminal cross-linked telopeptides of type II collagen)	Marker of cartilage degradation (Type II collagen breakdown)	Primarily studied in knee osteoarthritis	- Non-invasive (urine-based) - Strong correlation with disease progression - Demonstrated prognostic value in large cohorts	- Significant diurnal variation - Inter-individual and inter-ethnic variability - Not standardized for routine clinical use	Under investigation; not yet included in guidelines for OA diagnosis or management
**PINP** (Procollagen type I N-terminal propeptide)	Marker of bone formation (osteoblast activity and new collagen synthesis)	Monitoring therapy response in osteoporosis	- Low biological variability - Endorsed by IOF/IFCC as reference marker - Responds predictably to anabolic and anti-resorptive therapies	- Confounded by renal function (monomeric form) - Requires specific assay for intact (trimeric) PINP - Some inter-assay variability	Established in guidelines for osteoporosis monitoring and treatment response
**rs143383 (SNP in GDF5)**	Genetic variant associated with reduced GDF5 expression; modulated by DNA methylation	Associated with OA susceptibility (especially knee and hip OA)	- Mechanistically linked to OA development - Epigenetic modulation provides insight into gene-environment interactions	- Modest effect size (OR ~1.10) - Poorly validated in diverse ethnic groups - No clinically validated diagnostic thresholds	Experimental; not used in clinical practice; potential role in future risk panels

## Data Availability

No new data were created or analyzed in this study. Data sharing is not applicable to this article.
